# The Role of Preoperative Immunonutritional Scores in Predicting Complications After Subthalamic Nucleus Deep Brain Stimulation in Parkinson’s Disease

**DOI:** 10.3390/jcm14113811

**Published:** 2025-05-29

**Authors:** Marina Raguž, Marko Tarle, Petar Marčinković, Hana Chudy, Darko Orešković, Vladimira Vuletić, Tonko Marinović, Darko Chudy

**Affiliations:** 1 Department of Neurosurgery, Dubrava University Hospital, 10000 Zagreb, Croatia; petar.marcinkovic11@gmail.com (P.M.); darkoreskov@gmail.com (D.O.); tmarinovic79@gmail.com (T.M.); darko.chudy@gmail.com (D.C.); 2 School of Medicine, Catholic University of Croatia, 10000 Zagreb, Croatia; 3 Department of Maxillofacial and Oral Surgery, Dubrava University Hospital, 10000 Zagreb, Croatia; mtarle@sfzg.hr; 4 School of Dental Medicine, University of Zagreb, 10000 Zagreb, Croatia; 5 Department of Neurology, Dubrava University Hospital, 10000 Zagreb, Croatia; 6 Department of Neurology, Clinical Hospital Centre Rijeka, 51000 Rijeka, Croatia; vladimira.vuletic@gmail.com; 7 Department of Neurology, Faculty of Medicine, University of Rijeka, 51000 Rijeka, Croatia; 8 Medicine of Sports and Exercise Chair, Faculty of Kinesiology, University of Zagreb, 10000 Zagreb, Croatia; 9 School of Medicine, University of Zagreb, 10000 Zagreb, Croatia

**Keywords:** Parkinson’s disease, deep brain stimulation, HALP score, postoperative complications, AISI, SIRS, LMR, immunonutrition

## Abstract

**Background**: Parkinson’s disease (PD) is a progressive neurodegenerative disorder associated with systemic inflammation, immune dysregulation, and malnutrition, all of which may influence surgical outcomes. Subthalamic nucleus deep brain stimulation (STN DBS) is a widely used treatment for advanced PD, yet postoperative complications remain a concern. This study evaluates the predictive value of preoperative immunonutritional markers—including the Hemoglobin, Albumin, Lymphocyte, and Platelet (HALP) score, Aggregate Index of Systemic Inflammation (AISI), Lymphocyte-to-Monocyte Ratio (LMR), and systemic inflammatory response syndrome (SIRS)—for the risk of extracranial complications following STN DBS. **Methods**: A retrospective cohort study was conducted on 138 PD patients who underwent STN DBS. Clinical and laboratory data were analyzed to assess the association between preoperative immunonutritional markers and postoperative complications, including infections, wound healing disturbances, and surgical revisions. Logistic regression and receiver operating characteristic (ROC) analysis were performed to evaluate the predictive power of these markers. **Results**: SIRS emerged as the strongest predictor of complications (aOR = 6.99, 95% CI = 1.844–26.509), emphasizing the critical role of systemic inflammation in surgical outcomes. HALP, AISI, and LMR also demonstrated significant predictive potential, with HALP (AUC = 0.69) and LMR (AUC = 0.73) being the most robust predictors of complications. While albumin alone was not a significant predictor, it correlated with inflammatory markers and comorbidities, underscoring its role in broader risk assessments. **Conclusions**: This study underscores the value of preoperative immunonutritional markers in predicting complications following STN DBS in PD patients. Incorporating these markers into clinical risk stratification may enhance preoperative planning and personalized postoperative care, ultimately improving surgical outcomes. These findings, while promising, warrant validation through prospective, multicenter studies to refine predictive models and enhance patient outcomes.

^‡^ These authors also contributed equally to this work.

## 1. Introduction

Parkinson’s disease (PD) is a chronic and advancing neurodegenerative condition marked by a range of motor impairments and non-motor disturbances resulting from dopamine deficiency [1,2]. While levodopa remains the most effective pharmacological treatment, long-term dopaminergic therapy can lead to complications such as motor fluctuations and dyskinesias [3,4]. In addition to the well-established dopaminergic pathology, PD is increasingly recognized as a disorder involving systemic inflammatory and endocrine dysregulation. Microglial activation and elevated levels of pro-inflammatory cytokines such as IL-1β, IL-6, and TNF-α are implicated in neuronal degeneration and disease progression [5]. A recent meta-analysis further confirmed significantly elevated levels of these cytokines in the peripheral blood of PD patients, reinforcing the systemic nature of inflammation in PD [6]. In addition, dysfunction of the orexin system has been implicated in several aspects of PD pathogenesis, particularly concerning sleep–wake disturbances, autonomic dysregulation, and immune interactions [7]. For patients under 70 years of age, without dementia or psychosis, and with a favorable levodopa response, deep brain stimulation (DBS) targeting either the subthalamic nucleus (STN) or the globus pallidus internus (GPi) is a well-established surgical intervention that improves motor symptoms and reduces medication dependency. Over 244,000 DBS devices have been implanted worldwide, making it the most common surgical procedure for PD [1,2,3,4,8,9]. Despite its benefits, DBS is associated with potential complications, including procedure-related risks such as intracranial hemorrhage, as well as hardware-related issues like infection, electrode breakage, and skin erosion [10]. Although hardware complications are rarely life-threatening, they pose a significant clinical and financial burden [11]. Infection rates range from 2.6% to 9.3%, with a higher incidence following initial implantation than during revision procedures, likely due to surgical complexity [8,12]. Risk factors include male sex, diabetes mellitus, and increased BMI. Infections, typically caused by Gram-positive bacteria, are classified as superficial, deep, uncomplicated, or deep complicated (involving intracranial structures). Management strategies range from antibiotic therapy to partial or complete device removal, with full explanation associated with lower treatment failure rates [12]. Identifying patients at increased risk of complications is essential for optimizing surgical outcomes. This can be achieved using predictive scoring systems based on inflammatory and immunonutritional markers. Several immunonutritional and inflammatory biomarkers, including the Systemic Immune-Inflammation Index (SII), Neutrophil-to-Lymphocyte Ratio (NLR), Platelet-to-Lymphocyte Ratio (PLR), Aggregate Index of Systemic Inflammation (AISI), and the HALP score, have been evaluated across different clinical contexts for their ability to predict patient outcomes [13,14,15,16]. The SII, which integrates platelet, neutrophil, and lymphocyte counts, has been shown to predict the development of systemic inflammatory response syndrome (SIRS), a state of systemic inflammation defined by abnormal temperature, tachycardia, tachypnea, or leukocyte count alterations [17,18]. Previously, SIRS served as an early marker of inflammation, while nowadays it has been linked to adverse postoperative outcomes [19]. The AISI, incorporating neutrophils, lymphocytes, monocytes, and platelets, has demonstrated strong predictive value in sepsis and oncologic conditions [20,21]. Additionally, the HALP score, derived from hemoglobin, albumin, lymphocytes, and platelets, is widely used as an immunonutritional biomarker, with lower values correlating with poor outcomes in cancer and reconstructive surgery [22,23].

This study is the first to evaluate the prognostic potential of inflammatory and immunonutritional markers in predicting postoperative complications in PD patients undergoing STN DBS. By integrating these indices into clinical practice, risk stratification may be improved, allowing for tailored preoperative and postoperative care strategies that enhance patient outcomes.

## 2. Materials and Methods

This retrospective analysis was conducted using patient data retrieved from the electronic health records of the Department of Neurosurgery, Dubrava University Hospital in Zagreb, Croatia, a national referral center for stereotactic and functional neurosurgery. The study covered the period from 1 January 2010 to 31 December 2023 and included 138 patients diagnosed with Parkinson’s disease who underwent bilateral deep brain stimulation of the subthalamic nucleus. Electrode implantation was planned based on direct anatomical targeting using frameless MRI and CT images, acquired with a mounted Leksell frame (Elekta, Stockholm, Sweden). Devices used included Medtronic models 3387 and 3389 (Medtronic, Minneapolis, MN, USA), as well as Boston Scientific Vercise directional leads (Boston Scientific, Marlborough, MA, USA). Clinical and surgical data were obtained from the hospital’s digital documentation system. Laboratory parameters for calculating systemic immune scores were obtained from hospital admission laboratory findings. In addition, we extracted information regarding relevant comorbidities, including diabetes, hypertension, vascular pathology, and the use of antiplatelet or anticoagulant medications. Data on PD duration, symptomatology, and the presence of motor, cognitive, and autonomic impairments were also recorded [24]. Inclusion criteria were (a) PD patients qualified for STN DBS, operated and followed up on at our institution or a cooperative institution for at least a year, and (b) complete medical documentation. Exclusion criteria included incomplete medical documentation, and 8 patients were excluded based on this.

This study focuses on postoperative infections and wound-related complications following STN DBS in PD patients. It specifically examines extracranial complications, including skin erosions, localized infections, wound dehiscence, and abscess formation, while also considering hardware-related infections and their impact on wound healing and recovery.

Several systemic inflammation indices were computed using established formulas. The Aggregate Index of Systemic Inflammation (AISI) was derived by multiplying neutrophil, monocyte, and platelet counts, and dividing the product by the lymphocyte count: AISI = (neutrophils × monocytes × platelets)/lymphocytes [20]. The Systemic Immune-Inflammation Index (SII) was obtained by multiplying neutrophil and platelet counts and dividing by lymphocytes: SII = (neutrophils × platelets)/lymphocytes [13]. The Neutrophil-to-Lymphocyte Ratio (NLR) was calculated as the quotient of neutrophils and lymphocytes: NLR = neutrophils/lymphocytes [14]. The Platelet-to-Lymphocyte Ratio (PLR) was determined by dividing platelet count by lymphocyte count: PLR = platelets/lymphocytes [15]. The Lymphocyte-to-Monocyte Ratio (LMR) was obtained by dividing lymphocytes by monocytes: LMR = lymphocytes/monocytes [16]. These indices reflect the balance between inflammatory and immune components and can be linked to clinical outcomes in PD. The HALP score, which combines nutritional and hematologic markers, was calculated using the following formula: Hemoglobin (g/L) × Albumin (g/L) × Lymphocytes (/L) ÷ Platelets (/L) [23,25].

The study was conducted in accordance with the ethical standards outlined by the Ethics Committee of Dubrava University Hospital. All participants provided written informed consent, consistent with the principles set forth in the Declaration of Helsinki. The research protocol received formal approval from the Institutional Review Board of Dubrava University Hospital, Zagreb, Croatia (Reference: 2025/0128-10).

Statistical analyses were conducted using MedCalc software (version 12.5.0, MedCalc Software, Ostend, Belgium; https://www.medcalc.org, accessed on 16 March 2025), with outcomes illustrated in graphical form. Continuous variables are reported as mean values with corresponding standard deviations. The Kolmogorov–Smirnov test was employed to assess the distribution patterns of the variables, revealing non-uniform normality across parameters. Depending on data distribution, correlations between variables were evaluated using either Pearson’s or Spearman’s rank correlation coefficients. We also performed logistic regression to calculate adjusted odds ratios (aORs), while categorical data were compared using Chi-square and Fisher’s exact tests. The diagnostic performance of the parameters in predicting complications was evaluated through receiver operating characteristic (ROC) curve analysis. A *p*-value of less than 0.05 was considered statistically significant.

## 3. Results

**Figure 1:**
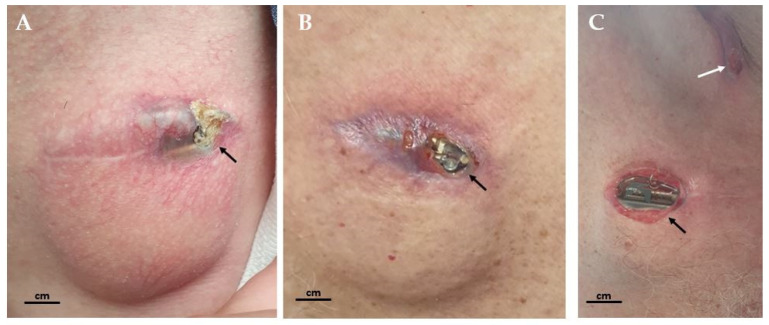
Extracranial skin infections and erosions after STN DBS. (**A**) Skin erosion and infection at the lateral part of the surgical incision, with skin thinning. The early stage of tissue breakdown is visible, with crust formation and local signs of inflammation. (**B**) Progression of skin erosion with exposure of the IPG through the surgical incision, accompanied by pronounced signs of local infection and an inflammatory reaction in the surrounding tissue. (**C**) Skin erosion with exposed IPG, showing significant local signs of infection. Additionally, skin erosion is present above the extensions in the medial supraclavicular area (white arrow), suggesting progressive soft tissue damage along the extension pathway. Black arrows indicate areas of inflammation, crust formation, and tissue breakdown surrounding the hardware.

In this study, 138 consecutive adult patients were enrolled, comprising 96 male patients (69.6%) and 42 female patients (30.4%), with a mean age of 61.32 ± 7.85 years (Table 1). Out of the total sample, 122 patients (89.7%) experienced no complications following DBS, while 14 patients (10.3%) encountered complications. The most frequent complication was infection, affecting 11 patients (7.97%), while other and hardware-related complications occurred in 3 patients (2.03%). Surgical revision was required in 12 patients (8.8%), with 33.3% of cases involving a single revision, while the majority (66.7%) required multiple interventions (Figure 1).

A statistically significant association was found between postoperative complications overall and the following variables: SIRS score (ρ = 0.348, *p* < 0.0001), HALP score (ρ = −0.198, *p* = 0.02), LMR (ρ = −0.243, *p* = 0.004), and albumin level (ρ = −0.183, *p* = 0.03). The AISI score demonstrated a trend toward correlation (ρ = 0.145, *p* = 0.09). Similarly, a statistically significant association was identified between the occurrence of infections and SIRS score (ρ = 0.248, *p* = 0.003), albumin (ρ = −0.262, *p* = 0.002), and LMR (ρ = −0.199, *p* = 0.01), while both AISI score (ρ = 0.142, *p* = 0.09) and HALP score (ρ = −0.142, *p* = 0.09) showed a trend toward statistical significance.

**Figure 2:**
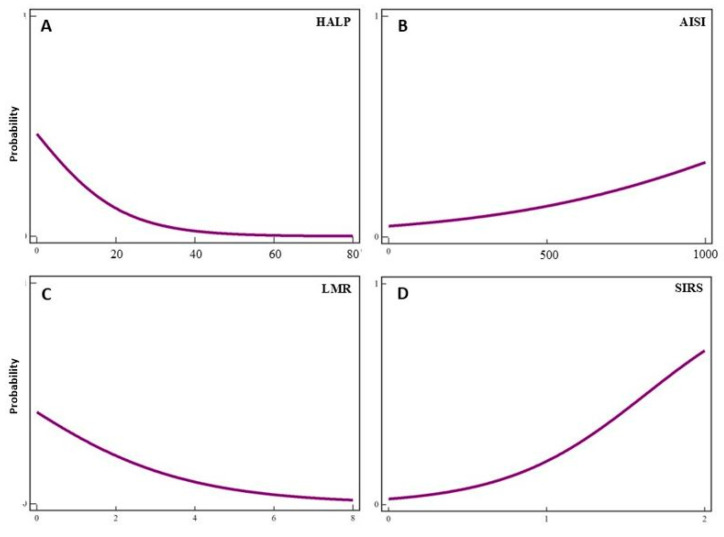
Logistic regression analysis demonstrates the statistically significant association between the occurrence of complications and preoperative immunological and nutritional markers. (**A**) The HALP score (ρ = −0.012, *p* = 0.01), (**B**) AISI score (ρ = −0.001, *p* = 0.008), and (**C**) LMR (ρ = −1.825, *p* = 0.02) show inverse relationships with complications, while (**D**) the SIRS score (ρ = 1.945, *p* = 0.004) exhibits a positive association, highlighting its strong predictive value for postoperative complications.

Logistic regression analysis revealed a statistically significant association between the occurrence of complications and preoperative HALP values (ρ = −0.012, *p* = 0.01), AISI score (ρ = −0.001, *p* = 0.008), LMR (ρ = −1.825, *p* = 0.02), and SIRS score (ρ = 1.945, *p* = 0.004) (Figure 2).

**Figure 3:**
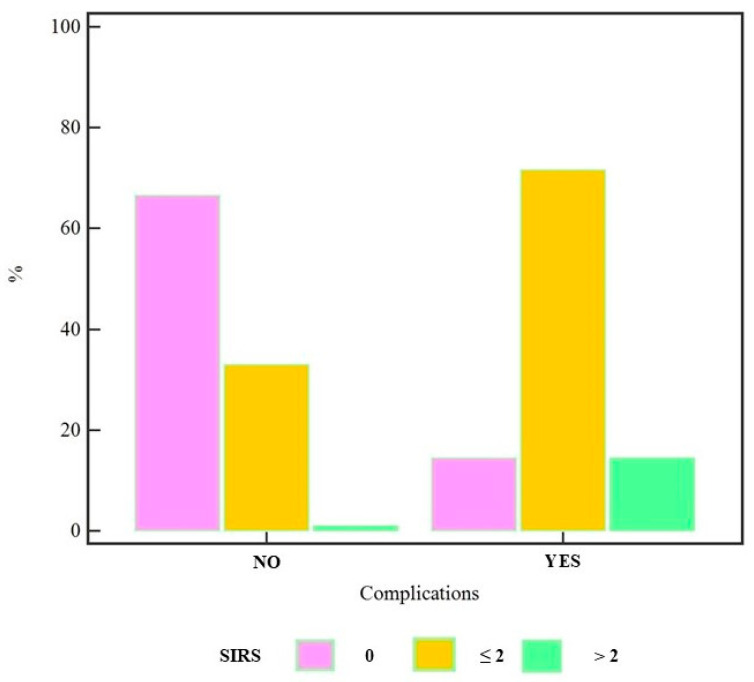
Results of the Chi-squared test showing a highly significant association between SIRS and complications (χ^2^ = 21.01, DF = 2, *p* < 0.0001), highlighting the strong correlation between elevated SIRS scores and the occurrence of complications, thus emphasizing its potential as a predictive marker.

In addition, the Chi-squared test demonstrated a highly significant association between SIRS and complications (χ^2^= 21.01, DF = 2, *p* < 0.0001) (Figure 3).

**Figure 4:**
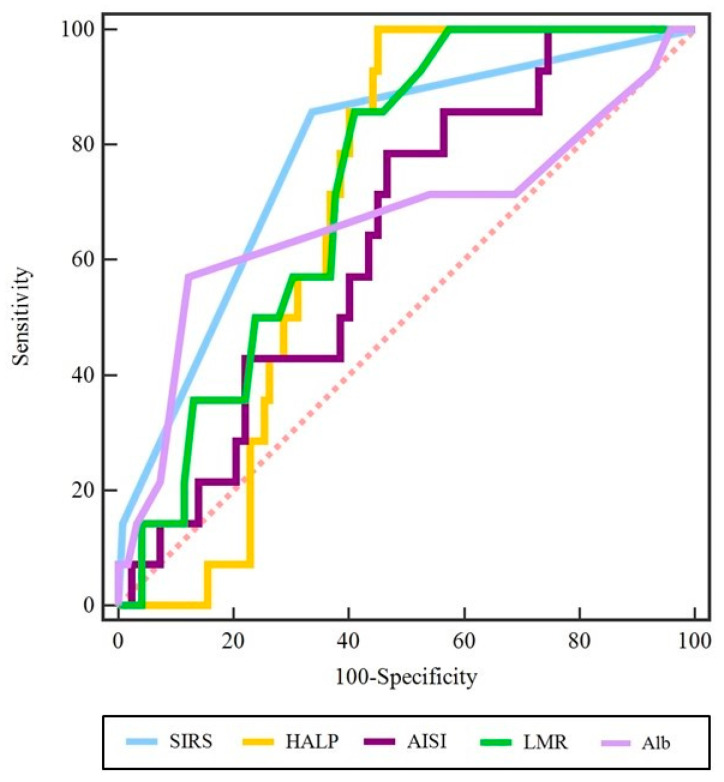
Receiver operating characteristic (ROC) analysis evaluating the predictive potential of preoperative scores for complications following STN DBS. The LMR (SE = 85.71, SP = 59.02, AUC = 0.73, J = 0.45, *p* < 0.0001) demonstrated the strongest predictive performance, while the HALP score (SE = 100.00, SP = 54.92, AUC = 0.69, Y = 0.55, *p* < 0.0001) showed moderate predictive potential. Moderate to low predictive capacity was also observed for the AISI score (AUC = 0.64, *p* = 0.04), SIRS score (AUC = 0.59, *p* = 0.003), and albumin level (AUC = 0.67, *p* = 0.07).

To evaluate the potential of these scores as clinical predictors of complications following STN DBS, ROC analysis was performed. The HALP score (SE = 100.00, SP = 54.92, AUC = 0.69, Y = 0.55, *p* < 0.0001) and LMR (SE = 85.71, SP = 59.02, AUC = 0.73, J = 0.45, *p* < 0.0001) emerged as strong predictors of complications. The other scores showed moderate predictive potential, including the AISI score (SE = 78.57, SP = 53.28, AUC = 0.64, J = 0.32, *p* = 0.04), SIRS score (SE = 20.00, SP = 97.59, AUC = 0.59, Y = 0.18, *p* = 0.003), and albumin level (SE = 57.14, SP = 87.70, AUC = 0.67, J = 0.45, *p* = 0.07) (Figure 4).

aOR analysis revealed that HALP, AISI, and LMR were associated with a slight but statistically significant reduction in the odds of complications. Specifically, for each unit increase in HALP, the odds of complications decreased by 0.99% (aOR = 0.99, 95% CI = 0.979–0.997), for AISI by 0.99% (aOR = 0.998, 95% CI = 0.997–0.999), and for LMR by 0.16% (aOR = 0.16, 95% CI = 0.033–0.778). Conversely, each unit increase in the SIRS score was associated with a 6.99% increase in the odds of complications (aOR = 6.99, 95% CI = 1.844–26.509).

Further analysis revealed significant correlations between clinical variables, comorbidities, and postoperative complications in PD patients undergoing STN DBS. Comorbidities were significantly associated with complications (ρ = 0.189, *p* = 0.028; Fisher’s exact test *p* = 0.045), while postoperative complications showed a strong correlation with diabetes (ρ = 0.213, *p* = 0.012) and a trend toward significance for vascular disease (ρ = 0.16, *p* = 0.06). Dehydration and infection were also strongly correlated (ρ = 0.286, *p* = 0.0007). Additionally, disease duration showed a trend toward a correlation with complications (ρ = 0.153, *p* = 0.0795), and significant associations were observed between sex and albumin levels (ρ = −0.261, *p* = 0.002) and between age and comorbidities (ρ = 0.264, *p* = 0.0019).

## 4. Discussion

To the best of our knowledge, this is the first study to investigate the prognostic potential of immunonutritional markers, including HALP, LMR, AISI, SIRS, and others for predicting postoperative extracranial complications in PD patients undergoing STN DBS. While these markers have been extensively studied in the context of malignancies and other surgical procedures, their role in assessing the risk of complications following DBS remains unexplored. By focusing on this specific patient population, our study provides novel insights into how preoperative immune and nutritional status may influence postoperative outcomes. These findings could pave the way for improved risk stratification and more personalized perioperative care, ultimately enhancing patient outcomes and reducing the burden of postoperative complications.

PD is not only a neurodegenerative disorder affecting motor function, but also causes metabolism, immunity, and nutrition imbalance. PD is closely associated with systemic inflammation, which significantly contributes to its pathophysiology and progression. Elevated levels of pro-inflammatory cytokines such as IL-6, TNF-α, and IL-1β have been observed in PD patients, indicating an active systemic inflammatory response [7,26,27,28]. Both central and peripheral inflammation play a role, with central processes involving activated microglia and astroglia, and peripheral inflammation originating from immune responses in the gut and bloodstream [29,30]. Gut-derived inflammation, driven by pro-inflammatory gut microbiota and impaired gut barrier function, further exacerbates systemic inflammation and its effects on the central nervous system [31,32].

Given the well-established role of inflammation and immune dysregulation in PD, along with its potential to exacerbate vulnerability to infections and complications, we were motivated to investigate the nutritional and immunological parameters, such as AISI, SII, SIRS, NLR, PLR, LMR, and HALP, as potential predictors of postoperative outcomes in patients undergoing DBS. While SII, NLR, and PLR did not demonstrate a significant correlation, HALP, SIRS, LMR, and AISI were significantly associated with the occurrence of postoperative complications. Furthermore, all indices that demonstrated significant correlations were also identified as significant predictors of postoperative complications.

SIRS and PD share common pathways involving systemic inflammation, which plays a critical role in PD pathogenesis and progression. Chronic inflammation in PD is characterized by elevated levels of pro-inflammatory cytokines, which are central to SIRS and have been observed in PD patients, contributing to neurodegeneration [29,30,31,32]. Furthermore, the systemic nature of SIRS, marked by widespread immune activation, mirrors the peripheral inflammatory responses seen in PD, including gut inflammation and immune dysregulation [32,33]. This connection suggests that the presence of SIRS or systemic inflammation may exacerbate neurodegeneration in PD or increase susceptibility to complications, particularly in surgical contexts like DBS, as shown in our results. Understanding the interplay between SIRS and PD could provide insights into optimizing perioperative care and addressing inflammation-driven disease mechanisms in PD patients.

Additionally, the HALP score is a composite immunonutritional biomarker that integrates several routinely collected laboratory indicators to assess a patient’s overall health status. It has been analyzed in various contexts and populations, primarily focusing on its prognostic capabilities in different diseases and conditions [22]. The HALP score has been studied across diverse fields, showing significant associations with demographic, socioeconomic, and health factors in the general population [33]. It has been a strong predictor of complications in head and neck reconstructive surgery [21] and a reliable prognostic marker in cancers such as hepatocellular carcinoma, non-Hodgkin lymphoma, and diffuse large B-cell lymphoma, where lower scores indicate poorer outcomes [34,35]. In stroke patients, lower HALP scores are linked to cognitive impairment and higher mortality [36], while in prostate cancer and glioblastoma, it serves as a cost-effective prognostic biomarker [22,37]. Chronic inflammation in PD raises pro-inflammatory cytokine levels, leading to reduced lymphocytes, anemia, and platelet dysregulation, while malnutrition, caused by dysphagia, decreased appetite, and medication side effects, lowers albumin levels as the liver shifts to acute-phase protein production [38,39,40]. Together, these disruptions highlight the HALP score as a valuable tool for assessing nutritional and inflammatory status and predicting complications in PD patients undergoing DBS. In our study, the HALP score emerged as a moderately strong predictor of postoperative complications.

The AISI score, a valuable marker for assessing systemic inflammation and predicting adverse outcomes in various medical conditions, demonstrated a significant correlation with complications and infections and emerged as a reliable predictor of postoperative complications. Higher AISI values are associated with adverse outcomes in obstetrics, COVID-19, and cardiovascular events [41,42,43]. The AISI, alongside indices like the SII, has broader applications in predicting poor survival outcomes in cancers and cardiovascular diseases [43,44]. Furthermore, the AISI is a promising marker for predicting the severity of odontogenic abscesses, outperforming traditional inflammatory indices [21]. Thus, the AISI reflects the balance between inflammatory and immune responses, making it a valuable tool for assessing systemic inflammation in PD, where chronic inflammation significantly impacts disease progression and overall health. As a non-invasive and cost-effective biomarker, the AISI provides valuable insights into a patient’s inflammatory status and potential preoperative risks, while also serving as a tool for predicting postoperative complications in PD, enabling optimized treatment strategies and improved clinical outcomes [45].

Similarly, the LMR showed a significant correlation with complications and was identified as a reliable predictor of postoperative complications. The LMR is a potential diagnostic marker for PD, with lower LMR levels associated with an increased risk of PD. While the LMR, along with other ratios like the NLR and AFR, shows relevance in PD diagnosis, it has lower sensitivity and specificity compared to the AFR when used alone [45]. The LMR is part of a growing set of hematological markers being studied to better understand and diagnose PD. Furthermore, a low LMR is consistently associated with an increased risk of complications across various conditions, particularly in cancer and cardiovascular diseases [16,46]. It is a prognostic marker for poor outcomes and can help predict complications, emphasizing its potential utility in clinical assessments and management strategies.

In our study, albumin, on its own, is not a significant predictor of postoperative complications in PD patients undergoing DBS. Although albumin is often linked to nutritional status and wound healing in some studies, it may not be a sufficiently strong factor for predicting complications in this context, especially when other predictors such as SIRS, HALP, and the AISI are considered. In addition, we demonstrated a well-established correlation between complication occurrence and comorbidities, especially diabetes and vascular diseases [47]. These findings emphasize the importance of addressing comorbidities and specific risk factors such as diabetes in the perioperative management of patients to minimize complications and improve surgical outcomes.

The implementation of immunonutritional markers into clinical workflows may provide a simple, cost-effective means of enhancing preoperative risk stratification in patients undergoing DBS. These scores, calculated from routinely available blood tests, could help identify patients at increased risk of postoperative complications. For these individuals, targeted interventions, such as early nutritional support, tighter glycemic control, individualized antibiotic prophylaxis, and enhanced postoperative surveillance, could be implemented to improve outcomes. This approach could be especially valuable in older patients or those with comorbidities, where inflammatory and nutritional status often fluctuate and standard risk assessment tools may fall short.

While this study provides valuable insights, several limitations should be considered. Its retrospective design limits the ability to establish causation, and the single-center setting may restrict the generalizability of the findings. Although the sample size was sufficient for initial analyses, the small number of patients in certain complication subgroups reduced the statistical power. Variability in patient characteristics could have introduced confounding factors. Additionally, while inflammatory and nutritional markers are valuable tools, external factors can influence their levels, potentially impacting their predictive accuracy. Another limitation of our study is the lack of detailed data regarding patient pharmacotherapy. As this was a retrospective analysis, medication records were not consistently structured or standardized across all participants, limiting our ability to assess the potential influence of specific drugs on immunonutritional markers. While most patients received standard dopaminergic therapy, it is possible that other medications, such as corticosteroids, anti-inflammatory agents, immunomodulators, or nutritional supplements, may have affected laboratory values such as lymphocyte counts, albumin levels, or inflammatory indices. Future prospective studies with more detailed pharmacological profiling are warranted to explore the interaction between specific medications and immunonutritional status in this patient population. Additionally, age and disease duration may represent confounding factors that could influence both immunonutritional profiles and postoperative outcomes. Although these variables were recorded and compared between groups, their complex interaction with systemic inflammation, nutritional depletion, and surgical risk was not the focus of this study. Further analyses with stratified or multivariate modeling may help to better elucidate their contribution. Importantly, this study does include long-term follow-up data, but further research in broader, multicenter cohorts is needed to confirm the findings and refine the predictive models. Addressing these limitations in future prospective studies could enhance our understanding of how systemic inflammatory and nutritional markers contribute to surgical outcomes in PD patients undergoing DBS.

## 5. Conclusions

This study highlights the critical role of systemic inflammatory and nutritional markers in predicting postoperative complications in PD patients undergoing DBS. Among the analyzed markers, SIRS emerged as the strongest predictor of complications, underscoring the significant impact of systemic inflammation on surgical outcomes. Additionally, the HALP score, AISI, and LMR demonstrated notable contributions to risk assessment, offering valuable insights into a patient’s inflammatory and nutritional status, which are critical factors in recovery and wound healing. Integrating these markers into routine clinical practice may involve calculating immunonutritional scores, such as HALP, AISI, LMR, and SIRS, from standard preoperative blood tests. Patients identified as high-risk based on these scores could benefit from targeted interventions, including preoperative nutritional optimization, proactive management of systemic inflammation, and enhanced perioperative monitoring. These strategies can reduce the incidence of complications, improve wound healing, and shorten recovery times, thereby improving the overall quality of care for PD patients undergoing DBS.

Further research is needed to validate and refine these predictive models, as well as to explore their utility in broader patient populations and surgical contexts. A more comprehensive understanding of how these markers interact with other clinical variables may also help develop robust, evidence-based guidelines for managing complex cases, ultimately improving surgical outcomes and long-term patient well-being.

## Figures and Tables

**Table 1:** Demographic, clinical, and laboratory data of PD patients with and without postoperative complications.

	Non-Complications	Complications	*p*-Value
Age, years	61.00 ± 7.93	64.50 ± 6.98	0.29
Sex, male/female	87/37	9/5	0.34
Duration of PD	10.00 ± 5.48	12.00 ± 4.76	0.08
Comorbidities, yes/no	58/64	11/3	0.57
Leukocytes (×10^9^/L)	6.45 ± 1.76	6.50 ± 1.64	0.98
Platelets (×10^9^/L)	246.50 ± 56.74	220.50 ± 45.65	0.06
Neutrophils (×10^9^/L)	4.24 ± 1.42	4.17 ± 1.08	0.27
Lymphocytes (×10^9^/L)	1.56 ± 0.45	1.61 ± 0.40	0.48
Monocytes (×10^9^/L)	0.39 ± 0.13	0.42 ± 0.13	0.99
SII	610.65 ± 355.79	692.33 ± 198.91	0.07
SIRS	0.40 ± 0.49	1.89 ± 0.55	0.001
NLR	2.89 ± 2.10	3.14 ± 0.87	0.08
PLR	100.04 ± 14.06	98.07 ± 22.37	0.17
AISI	232.76 ± 124.45	480.66 ± 225.96	0.002
LMR	5.80 ± 1.88	3.40 ± 0.71	0.004
NLR + PLR	132.10 ± 5.28	152.80 ± 21.96	0.08
HALP score	44.42 ± 18.91	34.83 ± 14.03	0.001
CRP	1.35 ± 2.24	2.15 ± 2.13	0.06

Data are presented as mean ± standard deviation. SII, Systemic Immune-Inflammation Index; SIRS, systemic inflammatory response syndrome; NLR, Neutrophil-to-Lymphocyte Ratio; PLR, Platelet-to-Lymphocyte Ratio; AISI, Aggregate Index of Systemic Inflammation; LMR, Lymphocyte-to-Monocyte Ratio; HALP score, Hemoglobin, Albumin, Lymphocyte, and Platelet score; CRP, C-reactive protein.

## Data Availability

All data generated or analyzed during this study are included in this published article.
